# Extract of *Herba Anthrisci cerefolii*: Chemical Profiling and Insights into Its Anti-Glioblastoma and Antimicrobial Mechanism of Actions

**DOI:** 10.3390/ph14010055

**Published:** 2021-01-12

**Authors:** Dejan Stojković, Danijela Drakulić, Marija Schwirtlich, Nemanja Rajčević, Milena Stevanović, Marina D. Soković, Uroš Gašić

**Affiliations:** 1 Department of Plant Physiology, Institute for Biological Research “Siniša Stanković”—National Institute of Republic of Serbia, University of Belgrade, Bulevar despota Stefana 142, 11000 Belgrade, Serbia; dejanbio@ibiss.bg.ac.rs (D.S.); mris@ibiss.bg.ac.rs (M.D.S.); 2 Institute of Molecular Genetics and Genetic Engineering, University of Belgrade, 11042 Belgrade, Serbia; danijeladrakulic@imgge.bg.ac.rs (D.D.); schwirtlich@imgge.bg.ac.rs (M.S.); milenastevanovic@imgge.bg.ac.rs (M.S.); 3 Faculty of Biology, University of Belgrade, 11000 Belgrade, Serbia; nemanja@bio.bg.ac.rs; 4 Serbian Academy of Sciences and Arts, 11001 Belgrade, Serbia

**Keywords:** *A. cerefolium*, extract, herba, phenolic composition, anti-glioblastoma, antimicrobial, mechanisms of action

## Abstract

*Anthriscus cerefolium* (L.) Hoffm. is a plant traditionally used around the globe since antiquity. Although widely used in many traditional medicines in different cultures, from the scientific point of view it is poorly investigated. Glioblastoma, a tumor type with poor prognosis, is the most common and lethal brain tumor in adults. Current therapeutic strategies for glioblastoma include surgery, radiation and chemotherapy. On the other hand, it has been revealed that patients with cancers are highly susceptible to microbial infections due to the invasive nature of cancer treatment approaches. This study was designed to investigate the chemical profile of *herba Anthriscii cerefoli* methanolic extract by applying UHPLC-LTQ OrbiTrap MS^4^ analysis and to analyze its anti-glioblastoma and antimicrobial activities. This study revealed that methanolic extract of *herba Anthrisc cerefolii* contained phenolic acids and flavonoids, with 32 compounds being identified. Anti-glioblastoma activity was investigated in vitro using A172 glioblastoma cell line. The cytotoxic effects of the extract on A172 cells were compared to the same effect on primary human gingival fibroblast (HGF-1) cells. Decreased rate of proliferation and changes in cell morphology were detected upon treatment of A172 cells with the extract. The antimicrobial activity of extract was tested against *Staphylococcus aureus* and *Candida* species. The extract was active against the tested bacterium and yeasts, inhibiting free floating cells and microbial biofilms. This study is the first one to provide a detailed description of the chemical profile of *A. cerefolium* extract dealing with scientific insights into its anti-glioblastoma and antimicrobial activities.

## 1. Introduction

According to the World Health Organization (WHO) grading of central nervous system (CNS) tumors, glioblastoma (GBM) is classified as a malignant grade IV glioma tumor [1]. With poor prognosis and a median survival time of about 15 months GBM is the most aggressive glioma and one of the deadliest forms of brain cancer [2,3]. In most cases by the time of diagnosis GBM is already widely spread. Existing therapeutic strategies for GBM include surgery, radiation and chemotherapy. Combined radiotherapy and chemotherapy are currently used for the cytoreduction of the tumor, but over 90% of patients usually experience rapid tumor recurrence [4,5,6]. Tumor recurrence, poor prognosis, and the side effects of radio- and chemotherapy suggest the need to prioritize the further search for novel therapeutic approaches, especially among natural sources.

It has been revealed that microbial infections contribute to cancer promotion through the production of carcinogenic metabolites and alterations in host physiological processes [7,8]. There is cumulative evidence that *Candida albicans* is able to stimulate the onset and progression of cancers by provoking inflammation and producing carcinogens such as acetaldehyde [9]. On the other side, bloodstream infections, no surgery, more than two hospitalizations, distant metastasis, absence of drainage tubes and radiotherapy are potential prognostic risk factors in cancer patients with *S. aureus* infection [10]. Therefore, the search for novel natural substances that can be used to prevent or treat cancer and related microbial infections is a high priority.

*Anthriscus cerefolium* (L.) Hoffm. is an annual aromatic plant, belonging to the chervil plant genus of the family Apiaceae. The genus encompasses twelve species, some of which are considered weeds [11]. For thousands of years, since the times of the ancient Greeks, the delicate young leaves of chervil have been used in spring tonics. Herbalists have used chervil throughout history as a diuretic, expectorant, digestive and skin freshener. It is also thought to relieve the symptoms of eczema, gout, kidney stones and pleurisy. Chervil is a traditional remedy for bad dreams, burns and stomach problems and supposedly, the whole plant alleviates hiccups. It is used as an eyewash for rinsing and refreshing the eyes [11,12]. Previous research regarding chemical composition of *Anthriscus* has been mainly focused on the flavonoids present in *A. sylvestris*. Thus quercetin, apigenin and rutin have been identified in *A. sylvestris* [13]. Furthermore, Dall’Acqua et al. [14] showed that fractions of this plant contain mainly luteolin-7-*O*-glucoside (cinaroside). 

Since herbalists have used chervil throughout history for numerous biological activities, one of the aims of the present study was to investigate the chemical composition of the methanolic extract of *herba Anthrisci cerefolii*. As traditional uses of the plant include its use against bad dreams, meaning that it probably contains compounds that can pass the blood-brain barrier, we investigated anti-glioblastoma activity of methanolic extract of *A. cerefolium.* Since the plant was traditionally used for the treatment of pneumonia, suggesting its antimicrobial effect, we tested the activities of extract against microbes *Staphylococcus aureus* and *Candida* species. 

## 2. Results and Discussion

### 2.1. Analysis of Phenolic Acids and Flavonoids

A review of the literature revealed that the chemical compounds derived from *Anthriscus cerefolium* have not been extensively studied. In the genus *Anthriscus*, the essential oils [15] and lignans [16] were the most investigated chemical compounds. Here we investigate the presence of phenolic acid and flavonoid derivatives in the methanolic extract of *herba Anthrisci cerefolii*. Since there is no literature data regarding these chemical compounds in *Anthriscus cerefolium*, we compared our results with results obtained in some other *Antriscus* species, as well as in other plants belonging to the Apiaceae family (Table 1).

Using liquid chromatography in combination with the hyphenated mass spectrometry technique, we identified 32 compounds in total. To the best of our knowledge, this is the first report in which 32 compounds are identified in this plant. Table 1 lists all identified compounds with retention times and major mass spectrometry characteristics (exacts masses, MS^2^, MS^3^, and MS^4^ fragmentations). Among 32 compounds, 17 of them belong to phenolic acids and related compounds, and the 15 of them are flavonoids aglycones and glycosides. The presence of 10 compounds (caffeic acid—**3**, aesculin—**4**, chlorogenic acid—**6**, *p*-coumaric acid—**7**, ferulic acid—**8**, rutin—**18**, cynaroside—**19**, apigetrin—**21**, luteolin—**24**, and apigenin—**26**) was confirmed by comparison with available standards. 

Regarding phenolic acid derivatives, in addition to simple acids, hexosides and esters with quinic acid were also found. All compounds, except *o*-hydroxybenzoic acid (compound **17**), belong to the group of hydroxycinnamic acids. Compound **17** found at 8.17 min and 137 *m*/*z* showed characteristic MS^2^ base peak at 93 *m*/*z* (generated by the loss of CO_2_—44 Da) and it has been previously detected in *Anthriscus vulgaris* Bernh. (Apiaceae) from Algeria [18]. Fragmentation pattern and MS spectra of compound **1** (dihydroxybenzoyl hexoside) is fully consistent with the literature [31]. As far as we know, this compound has not been detected so far in any extract of the plant from the Apiaceae family. Compounds **11** and **16** (eluted at 6.32 and 7.79 min, respectively) were marked as feruloylquinic acid isomers. In this case, we can also speculate about the position of esterification because Shrestha et al. [32] showed the differentiation of all feruloylquinic acid derivatives by MS^2^ base peaks. Namely, unlike other derivatives, MS^2^ base peak of 5-*O*-feruloylquinic acid was found at 191 *m*/*z*, so these compounds can be *cis* and *trans* isomers. Similarly, compounds **13**, **14**, and **15** (malonyl-dicaffeoylquinic acid isomers) found at 601 *m*/*z*, previously identified in *Erigeron breviscapus* (Apiaceae) extract, were named according to the available literature data [22].

**Figure 1:**
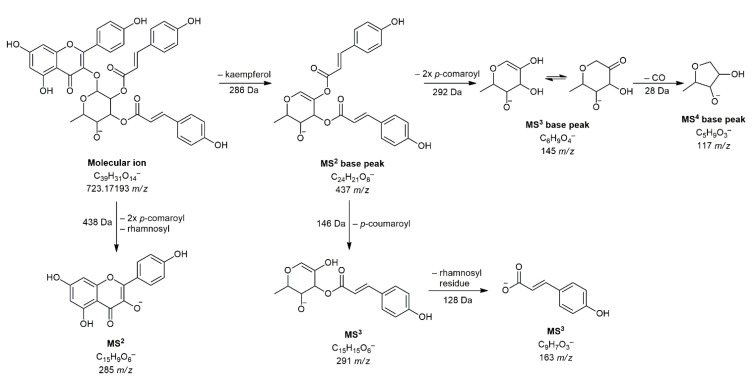
Schematic representation of fragmentation of compound **31** (kaempferol 3-*O*-(2″,3″-di-*p*-coumaroyl)-rhamnoside).

By analyzing flavonoids, compounds from the subgroup of flavonols and flavones were found. Only two aglycones were detected, luteolin (compound **24**) and apigenin (compound **26**) and both compounds have already been confirmed as constituents of *Anthriscus sylvestris* Hoffm. (Apiaceae) [25]. The most common compounds were flavonol glycosides with a *p*-coumaroyl residue. Almost all such compounds showed specific fragmentation where the mass of deprotected aglycone does not appear as MS^2^ base peak, but it is formed by loss of mass of aglycone. Thus, for example, compound **31** at 11.54 min and 723 *m*/*z* generated MS^2^ base peak at 437 *m*/*z*, which corresponds to the fragment resulting from kaempferol loss [M–H–286]^−^, while the secondary MS^2^ peak was found at 285 *m*/*z* (deprotonated kaempferol). Further, MS^3^ fragmentation gave a base peak at 145 *m*/*z* (loss of two *p*-coumaroyl residues) and secondary peaks at 291 and 163 *m*/*z* (Figure 1). Based on all these findings, a given compound is presumed to be kaempferol 3-*O*-(2″,3″-di-*p*-coumaroyl)-rhamnoside, which was previously isolated from the flowers of *Foeniculum vulgare* Mill. and *Foeniculum dulce* DC. (Apiaceae) [30]. 

**Figure 2:**
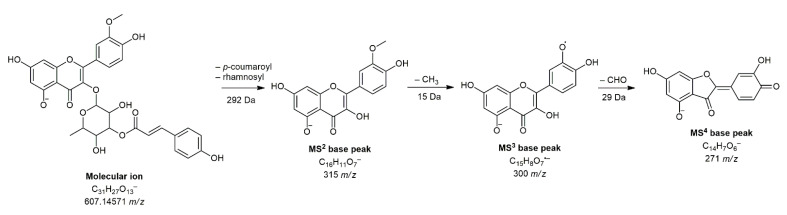
Proposed fragmentation pattern of compound **28** (isorhamnetin 3-*O*-(3″-*p*-coumaroyl)-rhamnoside).

Another compound that, to the best of our knowledge, has not been previously detected in any extract of the plants from the Apiaceae family is isorhamnetin 3-*O*-(3″-*p*-coumaroyl)-rhamnoside (**28**). The exact position of the *p*-coumaroyl residue can only be assumed, but the only compound corresponding to the exact mass and fragmentation of our compound was found in *Persicaria glabra* (Willd.) M.Gómez extract [33]. Its proposed structure and fragmentation are shown in Figure 2.

### 2.2. Anti-Glioblastoma Activity

Cytotoxic effect of the extract on A172 glioblastoma cell line was tested using crystal violet assay. Obtained result was compared with result achieved on human gingival fibroblast (HGF-1) cells. Results are presented in the Table 2. Extract concentration required for 50% inhibition of growth (IC50) of A172 glioblastoma cell line was 765.21 µg/mL. On the other hand, the extract displays no cytotoxicity on HGF-1 primary cells at concentrations up to 800 µg/mL.

Previous investigations showed that *A. cerefolium* extract had potent cytotoxic effect as determined by brine shrimp lethality assays on *Artemia* larvae [34]. Aqueous extract of *A. sylvestris* leaves showed no cytotoxicity towards RAW264.7 macrophages cells, but induced an anti-inflammatory defense response in this cell model [35]. Essential oil of the aerial parts of *A. caucalis* showed cytotoxic activity on liver hepatocellular carcinoma (HepG2) and human breast adenocarcinoma (MCF-7) cells [36]. Our results on *A. cerefolium* are supporting previous data regarding cytotoxicity against cancer cell lines of the species of the genus. However, this is the first report of the cytotoxic effects of *A. cerefolium* methanolic extract against a glioblastoma cell line.

**Figure 3:**
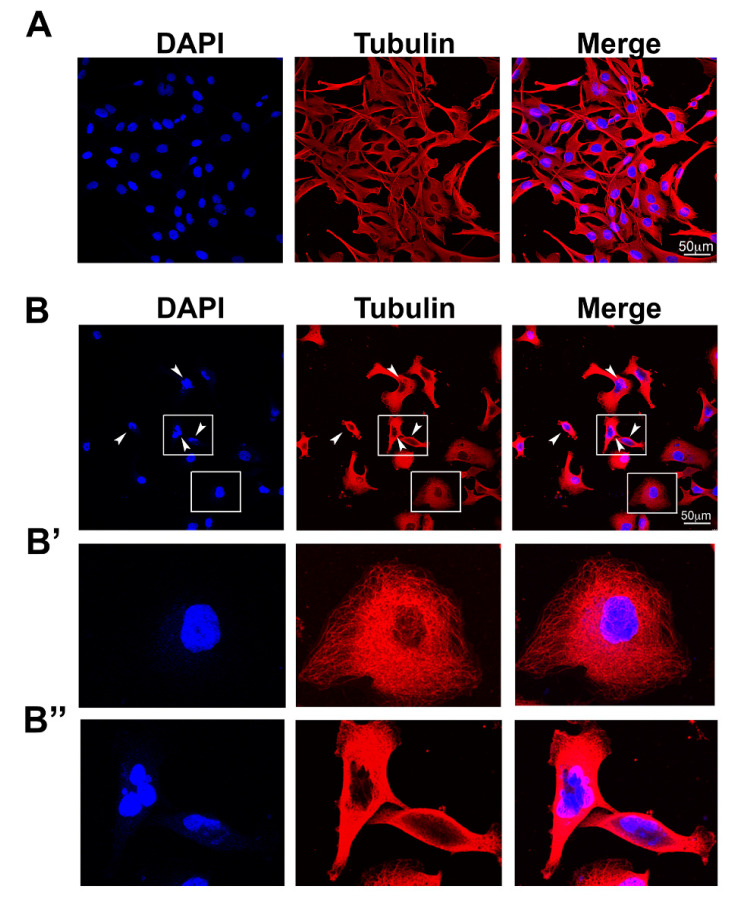
Morphology of A172 control cells (**A**) and A172 cells treated with *A. cerefolium* methanolic extract (**B**). Boxed regions in B are enlarged in (**B’**,**B’’**) figures. White arrowheads in B mark cells with multiple micronuclei.

The cell morphology of A172 glioblastoma cells treated with vehicle control or *A. cerefolium* methanolic extract (IC_50_ concentration) was analyzed by examining the expression of the cytoskeletal protein tubulin by immunofluorescence and laser confocal microscopy (Figure 3). As shown in Figure 3A, A172 control cells treated with vehicle (DMSO) exhibited a characteristic fibroblast-like morphology. The microtubule network is interconnected and appears filamentous (Figure 3A). In contrast, A172 cells treated with *A. cerefoilum* methanolic extract (Figure 3B) lost their fibroblast-like morphology became rounded with the fragmented nuclei. They exhibited a diffuse tubulin staining pattern and some of them contained multiple micronuclei (arrowheads in Figure 3B). Detected morphological changes are indicative for mitotic arrest and cell death (apoptosis). Further research is needed in order to dissect mechanism of action of the methanolic extract on A172 morphology.

**Figure 4:**
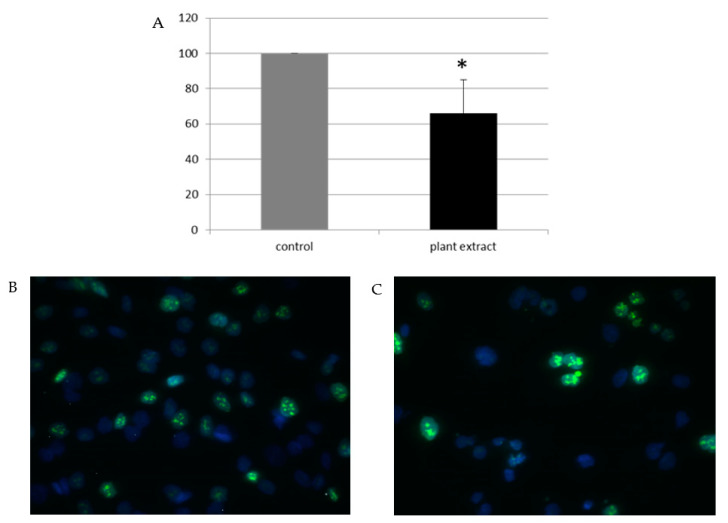
The effect of *A. cerefolium* methanolic extract on expression of Ki67 in A172 cell line; (**A**) The number of Ki67 positive cells in cells treated with the extract is presented as fold change of the number of Ki67 positive cells in cells treated with vehicle (DMSO) (arbitrarily set at 100%). Results are presented as the mean ± SD of three independent experiments, * *p* < 0.05 (**B**) A representative image of Ki67 immunostaining in the control cells treated with vehicle solvent DMSO; (**C**) A representative image of Ki67 immunostaining in cells treated with the extract. Nuclei are counterstained with DAPI.

The impact of the extract on the proliferation of A172 glioblastoma cells was investigated by analyzing the expression of Ki67 protein, a marker of proliferation. Ki67 is well-known marker for determination of the proliferation of tumor; its expression is used as a prognostic marker for cell proliferation in many tumors and it has been revealed that Ki67 value predicts the response to neoadjuvant chemotherapy [37]. The percentage of proliferating cells (Ki-67 labeling index) can discriminate more aggressive phenotypes of tumors; currently, thus the values of Ki67 labeling index is used both to predict the prognostic stratification of patients and to estimate the responsiveness to the resistance to chemotherapy [38]. By Ki67 immunostaining we detected that proliferation rate of A172 cells treated with the extract was decreased by aproximately 30% compared to proliferation rate of control cells (Figure 4). Our study is the first to explore the effect of *herba Anthrisci cerefolii* on the properties of glioblastoma cells. The obtained results provide a good basis for further research that could explore the possibilities of applying the extract of *herba Anthrisci cerefolii* in the treatment of glioblastoma.

It has been shown previously that quercetin, catechins and proanthocyanidins inhibit the proliferation of glioblastoma cells and induce their death; being able to cross the blood-brain barrier. Some of them inhibit pro-oncogene signaling pathways and intensify the effect of conventional anti-cancer therapies [39]. Our results indicate that extract of *herba Anthrisci cerefolii* was rich source of flavonoid molecules.

### 2.3. Antimicrobial Activities of Herba Anthrisci cerefolii

The antimicrobial activity of the *A. cerefolium* extract was tested by the microdillution method (Table 3). The most sensitive species to the effect of the *A. cerefolium* extract was yeast *C. tropicalis*, while the most resilient species to the microbicidal effect was the *S. aureus* ATCC 11632 bacterium strain. The results obtained for the extract were comparable with results obtained for the positive controls. It is important to highlight that extract was active against methicillin resistant strain of *S. aureus*.

Previous investigations were focused on essential oils from the genus *Anthriscus* and there are no reports about antimicrobial activity of methanolic extract of *A. cerefolium*. Namely, the essential oil from the aerial parts of *Anthriscus caucalis* M. Bieb showed significant activity against *Bacillus subtilis* and *Escherichia coli* with MIC values of 0.095 mg/mL and 0.105 mg/mL, respectively [36]. The essential oil obtained from the root of *Anthriscus nemorosa* (Bieb.) Sprengel showed significant activity against the following microbes: *Candida albicans*, *Staphylococcus epidermidis*, *Bacillus subtilis* and *Escherichia coli* [40]. 

The effect of the methanolic extract of *A. cerefolium* on *Staphylococcus aureus* (clinical isolate) biofilm formation was assessed by bacterial biofilm inhibition assay. The inhibition of biofilm formation by *S. aureus* was achieved at sub-MICs of the *A. cerefolium* extract. Bacterial biofilm was inhibited at 1/2 MIC for 69.88%, while at lower MICs inhibition capacity decreased (Table 4A). Regarding the inhibition of pre-formed yeast biofilms, the extract was equally active against all of the tested *Candida* species (Table 4B). Our results strongly point to the antibiofilm potential of the extract. These results are significant since microbial biofilms are more resistant to conventional therapeutics. According to our knowledge, there are no previous reports on antibiofilm potential of *Anthriscus* species.

**Figure 5:**
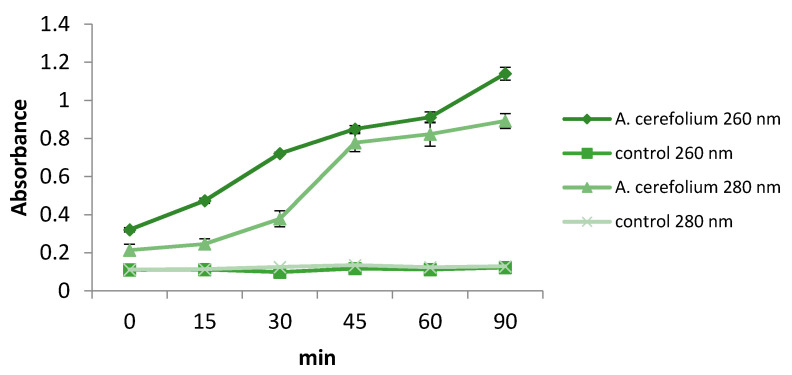
Leakage of cellular components in *C. albicans* measured at 260 nm and 280 nm.

Our results indicate that the extract of *A. cerefolium* did not significantly inhibit staphyloxanthin production in *S. aureus* at sub-MICs (data not shown). The extract had no influence on the ergosterol biosynthetic pathway in *C. albicans*, as well. The extract was able to induce leakage of cellular components in *C. albicans* (Figure 5), suggesting that the main mode of antifungal action is in the level of cell membrane permeability. Our results provide the first highlighting of the impact of *A. cerefolium* extract on the yeast cell membrane permeability.

## 3. Materials and Methods

### 3.1. Collection and Extraction of Plant Material

*Anthriscus cerefolium* (L.) Hoffm. (Apiaceae) was collected in Belgrade, Serbia, during the flowering period of plant in May 2018. The aerial parts of the plant were lyophilized and reduced to a fine powder. Plant material was successively extracted with methanol according to the procedure described previously [41,42].

### 3.2. Chemicals and Reagents

Solvents for UHPLC/MS analysis (acetonitrile and formic acid) were of MS grade, obtained from Fisher Scientific (Loughborough, UK). Ultrapure water was generated by deionization (Millipore, Billerica, MA, USA). Standards of phenolic acids and flavonoids (caffeic acid, aesculin, chlorogenic acid, *p*-coumaric acid, ferulic acid, rutin, cynaroside, apigetrin, luteolin, and apigenin) were purchased from Sigma-Aldrich (Steinheim, Germany).

### 3.3. UHPLC-LTQ OrbiTrap MS^4^ Analysis of Phenolic Acid and Flavonoid Derivatives

Analysis of compounds of interest was carried out by an Accela UHPLC system connected with LTQ OrbiTrap mass spectrometer equipped with heated-electrospray ionization (HESI) ionization applied in negative mode (ThermoFisher Scientific, Bremen, Germany). The separation was achieved using a Syncronis C18 column (100 × 2.1 mm, 1.7 μm particle size; ThermoFisher Scientific). The gradient elution program, settings of ion source and the other parameters of the mass detector were the same as previously described [43]. Identification of compounds was done according to their monoisotopic mass (obtained by full scan analysis) and MS^4^ fragmentation and also confirmed by literature data. Accurate mass of compounds was calculated using ChemDraw software (version 12.0, CambridgeSoft, Cambridge, MA, USA) and for instrument control, data acquisition and data analysis Xcalibur software (version 2.1, Thermo Fisher Scientific, Waltham, MA, USA) was used.

### 3.4. Investigation of the Antiproliferative Effect of A. cerefolium Extract

Crystal violet assay was used to study the cytotoxic effect of *A. cerefolium* extract on A172 glioblastoma cell line and human gingival fibroblasts cells HGF-1 (ATCC^®^ CRL-2014™). Cytotoxic effect of *A*. *cerefolium* methanolic extract on HGF-1 cells was determined as described by Stojkovic et al., [42]. The A172 cells were grown in high-glucose Dulbecco’s Modified Eagle Medium (DMEM) supplemented with 10% fetal bovine serum (FBS), 2 mM L-glutamine and 1% penicillin and streptomycin at 37 °C in 10% CO_2_. The day before treatment, 4 × 10^3^ cells were seeded per well in a 96-well plate. On the day of treatment, the fresh medium with different concentrations of the extract (250–1000 μg/mL) dissolved in dimethyl sulfoxide (DMSO) was added to the cells and cells were incubated with the extract for 48h. After that period, cells were washed twice with phosphate-buffered saline (PBS), stained with 0.5% crystal violet staining solution for 15 min at room temperature and after removal of crystal violet the cells were washed in a stream of tap water and left to air-dry at room temperature. Later, the dye was dissolved in methanol and the absorbance of dye was measured in a microplate reader Infinite 200 PRO at 590 nm. Control cells were treated with the same percentage of DMSO as treatment with the highest concentration of the extract. The DMSO concentration in the assay did not exceed 0.5%. The experiment was done in triplicate for each concentration of the extract and three independent experiments were performed. The results were expressed as IC50 values in μg/mL. 

### 3.5. Immunocytochemistry

Twenty four hours before treatment 2.5 × 10^4^ A172 cells were seeded per well on coverslips in a 12-well plate. After that, cells were treated with the extract (IC50 concentration) or vehicle DMSO (control cells) for 48 hours. Later, cells were fixed in 4% paraformaldehyde for 15 min at room temperature (RT), washed 3 times for 20 min in 1× PBS, permeabilized 10 min in 0.2% Triton X-100 in PBS and blocked for 1h at RT in 10% normal goat serum/1% bovine serum albumin (BSA) in PBS. Primary antibodies, rabbit anti-Ki67 antibody (diluted 1:250, Abcam, Cambridge, UK) and mouse anti-tubulin antibody (Abcam, diluted 1:100) were diluted in PBS containing 1% BSA/0.1% Triton X-100. After one hour incubation at room temperature with anti-Ki67 antibody, cells were washed 3 times for 15 min with 0.1% Triton X-100 in PBS and incubated with anti-rabbit secondary antibody conjugated with Alexa Fluor 488 (diluted 1:500 in 1% BSA/0.1% Triton X-100 in PBS) (Invitrogen, Life Technologies Corporation, Carlsbad, CA, USA) for 1 h. Afterward, cells were washed 3 times for 15 min with 0.1% Triton X-100 in PBS and stained with 0.1 mg/mL diaminophenylindole (DAPI; Sigma,, Saint Louis, MO, USA). Images were taken using a BX51 fluorescent microscope (Olympus, Tokyo, Japan) equipped with appropriate filters and analyzed using the Cytovision software (Applied Imaging Corporation, Santa Clara, CA, USA). Ki67 index was determined as number of Ki67 positive cells (without measurement of fluorescence intensity)/total number of cells. At least 10 independent images per experiment were chosen to score Ki67 positive cells in controls and at least 15 independent images per experiment were chosen to score Ki67 positive cells in treatments with the extract. Images for control and treatment were taken with the same magnification. In other experiments, after overnight incubation at 4°C with mouse anti-tubulin antibody, cells were washed 3 times for 15 min with 0.1% Triton X-100 in PBS and incubated for one hour at RT with biotinylated anti-mouse IgG antibody (diluted 1:500 in 1% BSA/0.1% Triton X100 in PBS, Vector Laboratories, Burlingame, CA, USA). Following washing 3 times for 15 min with 0.1% Triton X-100 in PBS, cells were incubated with DyLight 594 Streptavidin antibody (diluted 1:500 in PBS, Vector Laboratories) for one hour at RT. After washing 3 times for 15 min with 0.1% Triton X-100 in PBS, nuclei were stained with 0.1 mg/mL DAPI. Images were taken using a TCS SP8 confocal microscope (Leica Microsystems, Wetzlar, Germany) applying the Leica LAS AF-TCS SP8 software.

### 3.6. Antimicrobial Susceptibility Tests

The *A*. *cerefolium* methanolic extract at the concentration of 0.1–20 mg mL^−1^ (in 5% dimethylsulfoxide—DMSO) was tested for antibacterial and antifungal activity through the serial microdilution method as described previously [41,42]. All microorganisms were provided by the Institute for Biological Research “Sinisa Stanković”—National Institute of the Republic of Serbia, University of Belgrade (Belgrade, Serbia). *Staphylococcus aureus* (ATCC 11632), and methicillin-resistant *S. aureus* (MRSA isolate) were used for antibacterial analysis. For analysis of antifungal activity, the *Candida albicans* (ATCC 10231), *C. tropicalis* (ATCC 750), and *C. krusei* (clinical isolate) were used. Streptomycin and ampicillin were used as positive controls for antibacterial assays, whereas ketoconazole and bifonazole were selected as positive controls for the antifungal assays, while 5% DMSO was used as a negative control. The results were presented as minimum inhibitory concentration (MIC, required for microbial growth inhibition), bactericidal (MBC) and fungicidal concentrations (MFC), expressed in mg/mL.

### 3.7. Activity against Formation and Inhibition of Microbial Biofilms

The effect of *A. cerefolium* methanolic extract on *Staphylococcus aureus* (clinical isolate) biofilm formation was assessed by bacterial biofilm inhibition assay. Crystal violet was used as a staining solution for the biofilm and biofilm formation inhibition was evaluated by spectrophotometric techniques. Results were presented as percentage of biofilm formation inhibition with respect to untreated control [41].

The effect of *A. cerefolium* methanolic extract on inhibition of formed biofilm of *C. albicans*, *C. krusei* and *C. tropicalis* was determined as previously described [42]. MIC was defined as the minimum concentration of the extract that inhibited further growth of the initial biofilm, and minimum fungicidal concentration (MFC) represents the concentration of the extract that resulted in the level of luminescence presenting no fungal growth.

### 3.8. Insights into Modes of Antibacterial and Antifungal Actions of Extract

The staphyloxanthin inhibition assay in *S. aureus* was performed as previously described [41]. SubMIC concentrations of the extract, ranging from 1/2 MIC to 1/32 MIC, were used for the assay. The pigment production was measured at 465 nm using a spectrophotometer. The results were presented as percentage of staphyloxanthin production in treated bacterium with respect to non–treated control bacterium.

A *Candida albicans* strain was used for ergosterol binding and membrane permeability assays. In order to determine whether the antifungal effect of the extract was achieved via disruption of ergosterol biosynthetic pathway, serial dilutions (same as for microdilution method) of the extract were prepared with addition of ergosterol (25–100 µg/mL). After 24 h of incubation at 37 °C, MFC values were determined as explained for antifungal activity assay. The effect of *A. cerefolium* extract on cell membrane permeability was analyzed as described previously. The optical density of the filtrate was measured at room temperature (25 °C) at 260 and 280 nm (8453 spectrophotometer, Agilent, Santa Clara, CA 95051 United States) [42].

## 4. Conclusions

Methanolic extract obtained from *herba Anthrisci cerefolii* was investigated for the first time regarding its phenolic composition. Phenolic acids and their derivatives, phenolic- related compounds and flavonoids were identified in the extract. The extract decreased the proliferation rate of A172 glioblastoma cells and induced cell morphology changes indicative for mitotic arrest and apoptosis, while at the same time it was not cytotoxic to control HGF-1 cells. Additionally, the extract was active against bacteria and yeast, and the obtained results suggest that antifungal mode of action is associated with disruption of cell membrane permeability. Previously, it was published that some natural products, like green barley extract [44], and lactoferrin [45] exhibited an antiproliferative activity on cancer cells, making our results in agreement with literature pointing to the anticancer effects of natural products. Altogether, the obtained results serve as a good basis for further research of the anti-glioblastoma and antimicrobial mechanisms of actions of *herba Anthrisci cerefolii*.

## Figures and Tables

**Table 1:** High resolution mass spetrometry (HRMS) and MS^4^ data for phenolic acids and flavonoids identified in *Anthriscus cerefolium* extract.

Peak No.	Identified Compounds	*t*_R_, min	Molecular Formula, [M–H]^−^	Calculated Mass, [M–H]^−^	Exact Mass, [M–H]^−^	Δ ppm	MS^2^ Fragments, (% Base Peak)	MS^3^ Fragments, (% Base Peak)	MS^4^ Fragments, (% Base Peak)	Reference
Phenolic Acid Derivatives										
**1**	Dihydroxybenzoyl hexoside *^d^*	3.95	C_13_H_15_O_9_^−^	315.07216	315.07246	−0.31	109(10), 152(22), 153(100), 154(9), 268(10), 278(9), 279(23)	108(7), 109(100)	NA	/
**2**	Caffeoyl-hexoside isomer 1 *^c^*	4.61	C_15_H_17_O_9_^−^	341.08781	341.08750	0.31	135(4), 179(100), 180(3)	135(100)	79(21), 107(100), 117(49)	[17]
**3**	Caffeic acid *^a,b^*	4.61	C_9_H_7_O_4_^−^	179.03498	179.03487	0.11	89(19), 129(15), 134(11), 135(100), 143(23), 144(20), 161(13)	91(9), 94(64), 106(6), 107(100), 132(5)	NA	[18]
**4**	Aesculin *^a,c^*	4.88	C_15_H_15_O_9_^−^	339.07216	339.07142	0.74	177(100), 178(3)	89(4), 105(10), 133(100), 149(6)	89(100), 105(9), 123(15)	[17]
**5**	Caffeoyl-hexoside isomer 2 *^c^*	5.11	C_15_H_17_O_9_^−^	341.08781	341.08674	1.07	135(9), 179(100), 180(7), 295(3)	135(100)	79(18), 107(100)	[17]
**6**	5-*O*-Caffeoylquinic acid isomer 1 (Chlorogenic acid) *^a,b^*	5.24	C_16_H_17_O_9_^−^	353.08781	353.08753	0.28	179(3), 191(100)	85(95), 93(56), 111(41), 127(100), 171(32), 173(81)	83(11), 85(100), 97(10), 99(38), 109(29)	[19]
**7**	*p*-Coumaric acid *^a,c^*	5.29	C_9_H_7_O_3_^−^	163.04007	163.03968	0.39	99(13), 115(18), 116(12), 119(100), 128(16), 131(19), 135(12)	66(100), 91(60)	NA	[17]
**8**	Ferulic acid *^a,c^*	5.57	C_10_H_9_O_4_^−^	193.05063	193.05139	−0.76	111(10), 134(6), 145(5), 147(100), 148(15), 149(9), 150(7)	57(6), 85(13), 99(6), 101(7), 103(7), 119(7), 129(100)	55(20), 57(50), 73(8), 85(100), 101(14)	[17]
**9**	5-*O*-Caffeoylquinic acid isomer 2 *^b^*	5.69	C_16_H_17_O_9_^−^	353.08781	353.08795	−0.15	179(3), 191(100), 192(4)	85(98), 93(58), 109(22), 111(31), 127(100), 173(69)	81(4), 83(11), 85(100), 99(46), 109(27)	[19]
**10**	5-*O*-*p*-Comaroylqunic acid *^c^*	5.87	C_16_H_17_O_8_^−^	337.09289	337.09270	0.19	163(4), 173(8), 191(100)	85(96), 93(64), 109(26), 111(35), 127(100), 173(91)	81(17), 83(12), 85(100), 99(49), 109(59)	[20]
**11**	5-*O*-Feruloylquinic acid isomer 1 *^c^*	6.32	C_17_H_19_O_9_^−^	367.10346	367.10372	−0.26	191(100), 192(8), 193(4), 321(4)	85(100), 93(54), 109(25), 111(35), 127(93), 173(81)	57(100)	[17]
**12**	3,5-*O*-Dicaffeoylquinic acid *^c^*	7.04	C_25_H_23_O_12_^−^	515.11950	515.11763	1.87	191(13), 335(9), 353(100), 354(14)	179(4), 191(100)	85(93), 93(63), 111(32), 127(100), 173(76)	[21]
**13**	Malonyl-1,4-*O*-dicaffeoylquinic acid *^c^*	7.12	C_28_H_25_O_15_^−^	601.11989	601.12114	−1.25	395(55), 439(72), 440(12), 515(85), 516(20), 557(100), 558(24)	233(32), 335(4), 377(9), 395(100)	173(13), 233(100), 335(8)	[22]
**14**	Malonyl-1,5-*O*-dicaffeoylquinic acid *^c^*	7.34	C_28_H_25_O_15_^−^	601.11989	601.11856	1.33	233(10), 395(100), 396(13), 439(9), 515(5), 557(58), 558(10)	173(12), 233(100), 335(5)	155(3), 173(100)	[22]
**15**	Malonyl-4,5-*O*-dicaffeoylquinic acid *^c^*	7.47	C_28_H_25_O_15_^−^	601.11989	601.12119	−1.30	395(55), 396(11), 439(53), 515(56), 516(14), 557(100), 558(23)	233(30), 335(4), 377(10), 395(100), 515(3)	173(13), 233(100), 335(7)	[22]
**16**	5-*O*-Feruloylquinic acid isomer 2 *^c^*	7.79	C_17_H_19_O_9_^−^	367.10346	367.10270	0.75	191(100), 192(16), 321(17), 322(9), 323(9), 329(8), 330(13)	85(99), 93(45), 109(29), 127(100), 171(26), 173(56)	NA	[17]
**17**	*o*-Hydroxybenzoic acid *^b^*	8.17	C_7_H_5_O_3_^−^	137.02442	137.02435	0.06	93(100)	NA		[18]
Flavonoid derivatives										
**18**	Quercetin 3-*O*-(6″-rhamnosyl)-glucoside (Rutin) *^a,b^*	6.40	C_27_H_29_O_16_^−^	609.14611	609.14539	0.72	225(5), 271(7), 300(37), 301(100), 343(12)	151(77), 179(100), 255(45), 257(13), 271(76), 273(19)	151(100)	[19]
**19**	Luteolin 7-*O*-glucoside (Cynaroside) *^a,b^*	6.68	C_21_H_19_O_11_^−^	447.09329	447.08956	3.73	285(100), 286(13)	151(41), 175(100), 199(87), 217(80), 241(97), 243(70)	119(8), 131(86), 133(20), 147(100), 157(5)	[23]
**20**	Kaempferol 3-*O*-(6″-acetyl)-hexoside *^c^*	7.20	C_23_H_21_O_12_^−^	489.10385	489.10298	0.87	285(100), 286(9), 429(6)	151(37), 175(82), 199(88), 217(72), 241(100), 243(59)	185(49), 197(99), 198(100), 199(79), 213(61)	[24]
**21**	Apigenin 7-*O*-glucoside (Apigetrin) *^a,c^*	7.22	C_21_H_19_O_10_^−^	431.09837	431.09842	−0.05	268(11), 269(100), 270(11), 311(3)	149(30), 181(26), 183(27), 224(26), 225(100), 227(29)	157(35), 169(44), 181(63), 196(40), 197(100)	[17]
**22**	Kaempferol 3-*O*-rhamnoside *^c^*	7.65	C_21_H_19_O_10_^−^	431.09837	431.09750	0.87	255(6), 284(60), 285(100), 286(7), 327(4)	229(51), 241(29), 255(58), 256(51), 257(100), 267(45)	163(75), 185(14), 213(23), 229(100), 239(45)	[17]
**23**	Apigenin 7-*O*-(6″-acetyl)-hexoside *^c^*	8.43	C_23_H_21_O_11_^−^	473.10894	473.10989	−0.95	268(50), 269(100), 270(14), 311(6)	149(30), 151(21), 183(28), 197(34), 201(24), 225(100)	169(37), 181(57), 183(39), 196(21), 197(100)	[17]
**24**	Luteolin *^a,b^*	8.65	C_15_H_9_O_6_^−^	285.04046	285.03874	1.73	151(28), 175(72), 197(21), 199(69), 217(55), 241(100), 243(53)	197(100), 198(80), 199(59), 212(21), 213(44), 223(29)	153(4), 169(100), 179(10), 180(14), 182(5)	[25]
**25**	Kaempferol 3-*O*-(6″-*p*-coumaroyl)-hexoside *^c^*	9.39	C_30_H_25_O_13_^−^	593.13007	593.12989	0.18	285(100), 286(9), 307(31), 308(4)	151(100), 213(50), 229(57), 241(42), 243(36), 257(87)	83(4), 107(100)	[26]
**26**	Apigenin *^a,b^*	9.50	C_15_H_9_O_5_^−^	269.04555	269.04531	0.23	149(45), 151(29), 183(17), 201(27), 225(100), 226(18), 227(18)	169(13), 180(15), 181(100), 183(27), 196(20), 197(38)	117(17), 139(25), 152(100), 153(41), 163(7)	[25]
**27**	Kaempferol 3-*O*-(4″-*p*-coumaroyl)-rhamnoside *^c^*	9.90	C_30_H_25_O_12_^−^	577.13515	577.13701	−1.86	285(100), 286(9)	151(80), 229(38), 241(41), 255(33), 257(100), 267(27)	163(27), 211(7), 213(16), 229(100), 239(12)	[27]
**28**	Isorhamnetin 3-*O*-(3″-*p*-coumaroyl)-rhamnoside *^d^*	10.10	C_31_H_27_O_13_^−^	607.14572	607.14261	3.11	284(6), 285(27), 299(16), 300(17), 315(100), 316(11)	300(100)	151(21), 227(12), 255(61), 271(100), 272(51)	/
**29**	Quercetin 3-*O*-(2″,6″-di-*p*-coumaroyl)-hexoside *^c^*	10.50	C_39_H_31_O_16_^−^	755.16176	755.16248	−0.72	271(3), 285(4), 301(3), 307(6), 469(100), 470(20), 593(5)	135(11), 161(56), 179(100), 271(22), 307(68)	NA	[28]
**30**	Kaempferol 3-*O*-(2″,6″-di-*p*-coumaroyl)-hexoside *^c^*	11.08	C_39_H_31_O_15_^−^	739.16684	739.16671	0.14	285(9), 307(4), 453(100), 454(22), 455(4), 593(4)	135(10), 161(100), 163(31), 179(65), 289(12), 307(67)	117(3), 133(100)	[29]
**31**	Kaempferol 3-*O*-(2″,3″-di-*p*-coumaroyl)-rhamnoside *^c^*	11.54	C_39_H_31_O_14_^−^	723.17193	723.16845	3.48	285(53), 286(8), 437(100), 438(17), 439(3), 577(3)	145(100), 163(71), 187(24), 211(14), 273(29), 291(46)	117(100)	[30]
**32**	Kaempferol 3-*O*-[2″-(4‴-methoxycinammoyl)-6″-*p*-coumaroyl]-hexoside *^c^*	11.57	C_40_H_33_O_15_^−^	753.18249	753.18286	−0.36	285(100), 286(9), 315(39), 437(49), 453(12), 467(77), 468(12)	151(87), 185(47), 213(46), 229(60), 239(42), 257(100)	189(28), 213(57), 215(13), 229(100), 239(24)	[29]

*^a^* Confirmed by standards; *^b^* Earlier identified in some *Antriscus* species; *^c^* Identified in some other species from Apiaceae family; *^d^* For the first time identified in Apiaceae family; *t*_R_—retention time; Δ ppm—mean mass accuracy.

**Table 2:** Cytotoxic activity of *A. cerefolium* methanolic extract on A172 and HGF-1 cells.

Cell lines	*A. cerefolium* IC_50_ (µg/mL)
**A172**	765.21 ± 56.7
**HGF-1**	>800

**Table 3:** Antimicrobial activity of the *A. cerefolium* methanolic extract (mg/mL).

Bacteria		*A. cerefolium*	Streptomycin	Ampicillin
*S. aureus* (ATCC 11632)	MIC	2.50	0.17	0.34
	MBC	5.00	0.25	0.37
*S. aureus* MRSA	MIC	1.25	0.10	-
	MBC	2.50	-	-
**Yeasts**		***A. cerefolium***	**Ketoconazole**	**Bifonazole**
*C. albicans* (ATCC 10231)	MIC	1.25	0.50	0.15
	MFC	1.25	1.00	0.30
*C. krusei* (clinical isolate)	MIC	1.25	0.50	0.25
	MFC	1.25	1.00	0.50
*C. tropicalis* (ATCC 750)	MIC	0.62	0.30	0.25
	MFC	1.25	0.50	0.50

**Table 4:** The effects of *A. cerefolium* methanolic extract on: (**A**) bacterial biofilm formation by *S. aureus* at sub-MICs (%) and (**B**) on MIC and MFC (mg/mL) in formed fungal biofilm.

(A) Inhibition of *S. aureus* bacterial biofilm formation					
	1/2 MIC	1/4 MIC	1/8 MIC	1/16 MIC	1/32 MIC
*A. cerefolium*	69.88 ± 6.86	67.91 ± 4.99	44.25 ± 4.70	NI	NI
Streptomycin	55.64 ± 2.12	35.33 ± 1.47	33.22 ± 1.08	15.21 ± 1.12	NI
**(B) Inhibitiory and fungicidal effects on formed fungal biofilms**					
**Fungi**		***A. cerefolium***		**Fluconazole**	
		**MIC**	**MFC**	**MIC**	**MFC**
*C. albicans* (ATCC 10231)		5.00	10.00	8.00	9.00
*C. krusei* (clinical isolate)		5.00	10.00	2.00	3.00
*C. tropicalis* (ATCC 750)		5.00	10.00	3.00	6.00

## Data Availability

All data is presented in the manuscript.
